# Comparison of neoadjuvant chemotherapy response and prognosis between HR-low/HER2-negative BC and TNBC: an exploratory real-world multicentre cohort study

**DOI:** 10.3389/fendo.2024.1347762

**Published:** 2024-03-19

**Authors:** Jing Peng, Yue Hong, Qitong Chen, Feng Xu, Danhua Zhang, Jia Yao, Qiongyan Zou, Liqin Yuan, Lun Li, Qian Long, Liqiu Liao, Mingwen Liu, Xuan Liu, Shouman Wang, Wenjun Yi

**Affiliations:** ^1^ Department of General Surgery, The Second Xiangya Hospital, Central South University, Changsha, China; ^2^ Clinical Research Centre For Breast Disease In Hunan Province, Changsha, China; ^3^ Department of Breast Surgery, Xiangya Hospital, Central South University, Changsha, China; ^4^ Department of Breast Surgery, the First People's Hospital of Xiangtan City, Xiangtan, Hunan, China

**Keywords:** breast cancer, estrogen receptor, progesterone receptor, triple-negative breast cancer, HR-low/HER2-negative breast cancer, neoadjuvant chemotherapy

## Abstract

**Objective:**

Hormone receptor (HR)-low/HER2-negative breast cancers (BCs) are more likely to be basal-like BCs, with similar molecular features and gene expression profiles to HR-negative (estrogen receptor <1% or negative and progesterone receptor <1% or negative) BCs. Recently, with the clinical application of adjuvant intensive therapy for triple-negative breast cancer (TNBC), the prognosis of TNBC patients without pathological complete response (pCR) has significantly improved. Therefore, it is necessary to reanalyse the prognostic characteristics of clinically high-risk HR-low/HER2-negative BC.

**Methods:**

According to the inclusion and exclusion standards, 288 patients with HR-low/HER2-negative BC and TNBC who received NAC and were followed up between 2015 and 2022 at three breast centres in Hunan Province, China, were enrolled. Inverse probability of treatment weighting (IPTW) was utilized to mitigate imbalances in baseline characteristics between the HR-low/HER2-negative BC group and TNBC group regarding event-free survival (EFS) and overall survival (OS). The primary clinical endpoints were pCR and EFS, while the secondary endpoints included OS, objective response rate (ORR), and clinical benefit rate (CBR).

**Results:**

The pCR rate (27.1% vs. 28.0%, *P* = 1.000), ORR rate (76.9% vs. 78.3%, *P* = 0.827) and CBR rate (89.7% vs. 96.5%, *P* = 0.113) after NAC were similar between the HR-low/HER2-negative BC and the TNBC group. EFS in patients with non-pCR from the 2 groups was significantly inferior in comparison to patients with pCR (*P* = 0.001), and the 3-year EFS was 94.74% (95% CI = 85.21% to 100.00%) and 57.39% (95% CI =43.81% to 75.19%) in patients with pCR and non-pCR from the HR-low/HER2-negative BC group, respectively, and 89.70% (95% CI = 82.20% to 97.90%) and 69.73% (95% CI = 62.51% to 77.77%) in the TNBC patients with pCR and non-pCR, respectively.

**Conclusions:**

In the real world, the therapeutic effects of NAC for HR-low/HER2-negative BCs and TNBCs were similar. EFS of patients with non-pCR in the HR-low/HER2-negative BC group was inferior to that of the TNBC group with non-pCR, suggesting that it is necessary to explore new adjuvant intensive therapy strategies for these patients.

## Introduction

Breast cancer (BC) serves as the foremost contributor to cancer-related fatalities among women (1). Based on the immunohistochemistry (IHC) expression of the estrogen receptor (ER), progesterone receptor (PR), and human epidermal growth factor receptor 2 (HER2), BCs can be categorized into 4 distinct molecular subtypes as follows: HER2 overexpression, luminal A, luminal B, and triple-negative breast cancer (TNBC) (2).

Regarding clinical decisions and prognosis, the expression of hormone receptors (HR), which includes ER and PR, stands as one of the most critical biomarkers for breast cancer (BC) patients. The 2010 American Society of Clinical Oncology/College of American Pathologists (ASCO/CAP) guidelines suggest that BCs with ER/PR expression of ≥1% should be regarded as HR positive (3). Moreover, the prognosis has remarkably benefited from endocrine therapy for the HR-positive BC patients (4). Recently, however, it has been reported that the efficacy of endocrine therapy in HR-low-expression BC remains uncertain (5–10). The 2020 ASCO/CAP guidelines recommend considering HR-low/HER2-negative BCs to be characterized by HER2-negative status and 1-10% ER/PR expression. They are resemble to basal-like BCs (11), with similar molecular features, gene expression profiles and incidence of BRCA 1/2 mutation to those of HR-negative (ER negative or <1% and PR negative or <1%) BCs (8, 12, 13).

It has been reported that the efficacy of neoadjuvant chemotherapy (NAC) and the survival of patients with HR-low/HER2-negative BCs and patients with TNBCs after NAC are similar (7). An analysis of neoadjuvant clinical trials (14) involving the randomized clinical trials of GeparQuinto (n=2491) (15, 16) and GeparSepto (n=1206) (17) from the German Breast Group (GBG) and Arbeitsgemeinschaft Gynäkologische Onkologie, Breast Group (AGO-B), found no statistically significant difference in the pathological complete response (pCR) rate between patients with HR-low/HER2-negative BC and those with TNBC (OR=1.47, 95% CI=0.89 to 2.42, *P* = 0.132). The same result was also observed in disease-free survival, distant disease-free survival, and overall survival (OS) between these 2 groups.

In recent years, the improvements of NAC and the proposal of an adjuvant intensive therapy strategy have significantly improved the prognosis of TNBC (18). The CREATE-X, SYSUCC-001 Randomized Clinical Trial provided the basis for the application of capecitabine in non-PCR patients with TNBC (19–21). However, adjuvant intensive therapies for HR-low/HER2-negative BCs have not been sufficiently studied. Therefore, it is necessary to reanalyse the prognostic characteristics of clinically high-risk HR-low/HER2-negative BCs, especially in non-pCR patients after NAC.

This real-world multicentre study intended to compare baseline characteristics, efficacy of NAC, and survival outcomes between patients with TNBCs and patients with HR-low/HER2-negative BCs.

## Methods

### Study design

**Figure 1:**
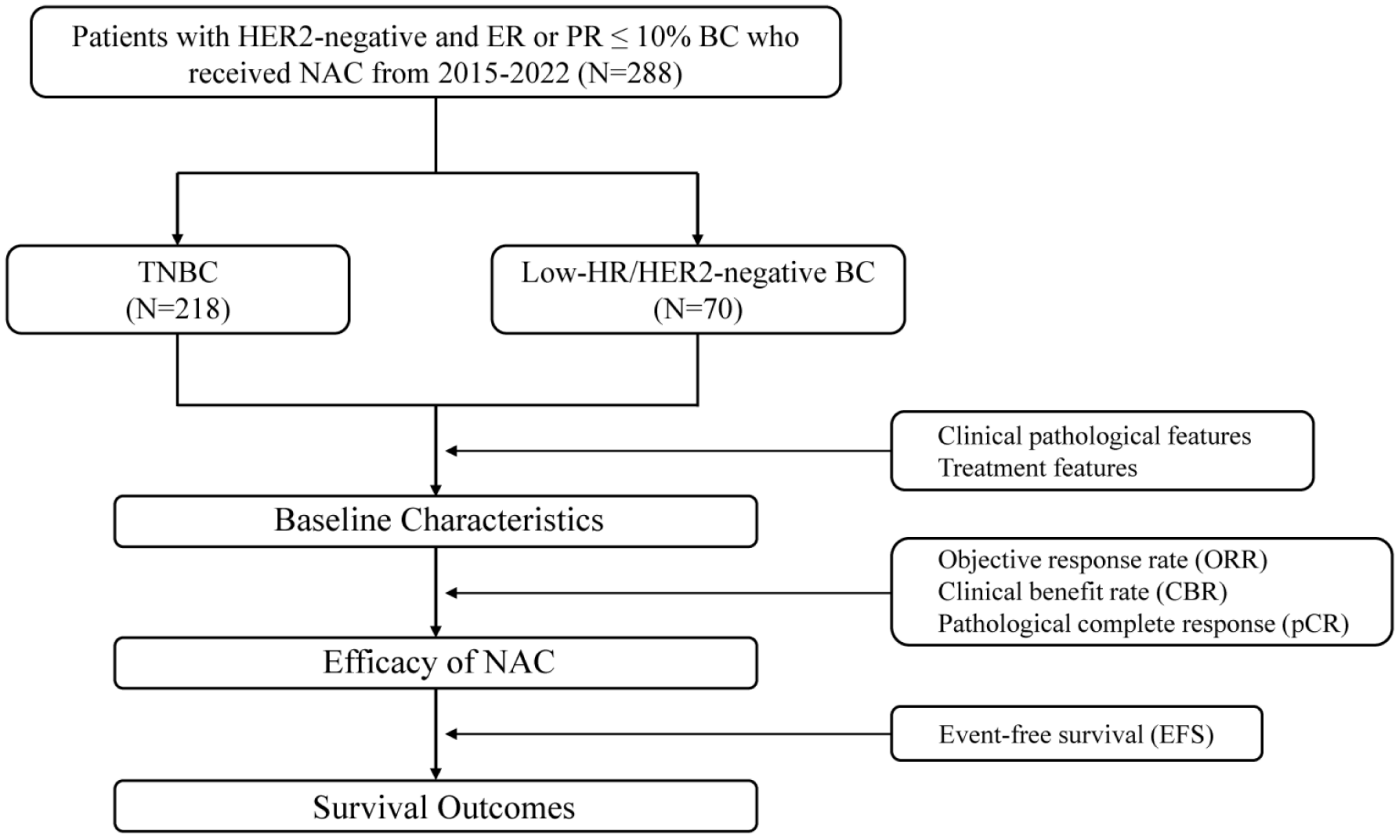
Trial design.

This was a retrospective multicentre real-world observational cohort study. This study included 288 BC patients from the Department of Breast Surgery of the Second Xiangya Hospital, Changsha, Hunan, China; Department of Breast Surgery of Xiangya Hospital, Changsha, Hunan, China; and the Department of Breast Surgery of the First People’s Hospital of Xiangtan City, Hunan, China, from 2015 to 2022. Patients were divided into 2 groups: HR-low/HER2-negative BC group (70 cases) and TNBC group (218 cases). We conducted follow-up assessments, collected clinical data, and compared the treatment efficacy and clinical outcomes of the 2 groups of patients (**Figure 1**).

### Participants

Patients were included based on the following criteria: (1) female sex; (2) age of 20-80 years; (3) stage II or III; (4) histologically confirmed primary HER2-negative BC (IHC score of 0 or 1, or 2 with a negative result on fluorescence *in situ* hybridization) (20); (5) ER or PR ≤ 10% (the pathological results that contained a ER and PR percentage value IHC staining were adopted); and (6) received NAC. We strictly adhered to the following exclusion criteria: (1) unclear or missing ER or PR expression data; (2) advanced and metastatic BC; (3) inflammatory or bilateral BC; and (4) incomplete follow-up data. This study was conducted after approval at the ethics committee of the Second Xiangya Hospital of Central South University.

HR-low/HER2-negative BCs were defined as ER-low positive (1-10%) with PR-low positive (1-10%) or negative, PR-low positive with ER-low positive or negative, and HER2-negative status. ER, PR, and HER2 status were assessed by the pathology departments of the respective centres.

We extracted age, menopausal status (postmenopausal/premenopausal), ER expression percentage, PR expression percentage, clinical stage (T1-2/T3-4), pathological lymph node status (N0/N+), histological type (IDC/Other), tumour grade (II, III, unknown), NAC cycles (<6/≥6) and regimen (AC-T, TAC, Other), platinum-based NAC regimen (yes/no), breast surgery (breast-conserving surgery/mastectomy), axilla surgery (ALND/SLNB), therapeutic evaluation (Overall Response Rate/ORR, Clinical Benefit Rate/CBR, pCR), event-free survival (EFS) and OS from the database. Staging was evaluated according to the American Joint Committee on Cancer (AJCC) classification.

### Clinical endpoints

The primary endpoints were the pCR rate and EFS. pCR was identified as the absence of invasive BC tumours in the breast and axillary lymph nodes surgical specimens after NAC (ypT0/is ypN0). EFS was described as the duration from the start of NAC until the first occurrence of any of the following events: disease progression without surgical treatment, local or distant recurrence, mortality from any cause, etc.

The secondary endpoints were OS, ORR and CBR, which were evaluated based on response evaluation criteria in solid tumours (RECIST 1.1). OS was described as the duration from the start of NAC until mortality from any cause. The ORR was characterized as the proportion of patients with complete response and partial response, while the CBR was determined by those with complete response or partial response or stable disease (22, 23). Ultrasonography and/or magnetic resonance imaging (MRI) were used for disease assessments at baseline, once every 2 or 3 cycles of NAC and once until surgery.

### Statistical analysis

Continuous variables were summarized using the mean, while categorical variables were presented using frequency. T tests were employed to assess the differences in age between the HR-low/HER2-negative BC and TNBC groups, and the chi-square test was applied to compare the clinicopathological data, ORR, CBR and pCR between the two groups. A logistic regression model was employed to explore the association between each variable and pCR. Cox regression was employed to model EFS in both groups. The original distribution of demographic and clinicopathological characteristics between the HR-low/HER2-negative BC and TNBC groups were evaluated by the standardized mean difference (SMD); a value >10% indicated an unbalanced distribution between the two groups. Inverse probability of treatment weighting (IPTW) was conducted to balance baseline characteristics between the HR-low/HER2-negative BC and TNBC groups (24) (**Table 1**). The weights were calculated as the inverse of propensity scores, defined as the predicted probability of subtypes on age, menopausal status (postmenopausal/premenopausal), clinical stage (T1-2/T3-4), pathological lymph node status (N0/N+), tumour grade (II, III, unknown), and histological type (IDC/Other), applying a nonparametric covariate balancing method to estimate propensity scores (25). Highly represented characteristic assignments were weighted less, and rarer characteristic assignments were weighted more. The SMD was utilized again to assess balance, and an SMD within 10% was considered acceptable, while 0% was considered ideal (24). EFS and OS were evaluated with Kaplan−Meier survival curves and the clog log transformation (26). All statistical tests were two-sided, and significance was defined as a *P* value < 0.05. All analyses were conducted using R 4.2.2.

**Table 1:** Baseline patient characteristics^a^.

Characteristic	Unweighted population			IPTW-weighted population		
	Low-HR/ HER2-negative	TNBC	SMD	Low-HR/ HER2-negative	TNBC	SMD
	N=70 (%)	N=218 (%)		N=287.5 (%)	N=288.03 (%)	
Age, year (mean(SD))						
	48.37 (9.87)	47.59 (9.72)	0.080	47.59 (9.63)	47.75 (9.71)	0.016
Menopausal status						
Postmenopausal	31 (44.3)	85 (39.0)	0.108	110.0 (38.3)	115.5 (40.1)	0.038
Premenopausal	39 (55.7)	133 (61.0)		177.4 (61.7)	172.5 (59.9)	
Clinical tumour stage						
T1-2	50 (71.4)	141 (64.7)	0.145	191.7 (66.7)	191.1 (66.4)	0.007
T3-4	20 (28.6)	77 (35.3)		95.7 (33.3)	96.9 (33.6)	
Pathological nodal status						
N0	22 (31.4)	80 (36.7)	0.111	94.0 (32.7)	101.6 (35.3)	0.055
N+	48 (68.6)	138 (63.3)		193.4 (67.3)	186.4 (64.7)	
Histological tumour type						
IDC	66 (94.3)	195 (89.4)	0.178	258.3 (89.9)	260.9 (90.6)	0.024
Other	4 (5.7)	23 (10.6)		29.1 (10.1)	27.2 (9.4)	
Tumour grade						
II	38 (54.3)	103 (47.2)	0.251	144.9 (50.4)	141.5 (49.1)	0.031
III	27 (38.6)	83 (38.1)		105.0 (36.5)	109.5 (38.0)	
Unknown	5 (7.1)	32 (14.7)		37.5 (13.1)	37.0 (12.9)	

a Low-HR/HER2-negative, low hormone receptor/human epidermal growth factor receptor 2 negative; TNBC, triple-negative breast cancer; SMD, std mean difference; SD, standard deviation; IDC, invasive ductal carcinoma.

## Results

### Baseline patient characteristics

The HR-low/HER2-negative BC and TNBC groups were composed of 70 and 218 patients, respectively. The mean age at first diagnosis was 48.37 years in the HR-low/HER2-negative BC group and 47.59 years in the TNBC group. Females were more frequently premenopausal in both groups (HR-low/HER2-negative BC: 55.7%; TNBC: 61.0%). Most patients had T1 or T2 tumours (HR-low/HER2-negative BC: 71.4%; TNBC: 64.7%), positive nodal status (HR-low/HER2-negative BC: 68.6%; TNBC: 63.3%), and invasive ductal carcinoma (HR-low/HER2-negative BC: 94.3%; TNBC: 89.4%). Grade 2 and 3 disease accounted for 54.3% and 38.6% of the total number of patients in the HR-low/HER2-negative BC group, and in the TNBC group, 47.2% and 38.1% of patients had tumours classified as grade 2 and 3, respectively. After IPTW adjustment, the covariates between the HR-low/HER2-negative BC and TNBC groups were found to be homogenous (**Table 1**).

The median number of treatment cycles for all 288 eligible participants was 6. A total of 65.7% of patients in the HR-low/HER2-negative BC group and 65.1% of patients in the TNBC group received no less than 6 cycles of NAC. The majority of patients received anthracycline-taxane-based NAC (HR-low/HER2-negative BC: 94.3%; TNBC: 89.0%), and only a small percentage of patients were treated with platinum (HR-low/HER2-negative BC: 12.9%; TNBC: 17.9%). After neoadjuvant therapy, most patients underwent mastectomy (HR-low/HER2-negative BC: 90.0%; TNBC: 96.3%) or axillary lymph node dissection (ALND) (HR-low/HER2-negative BC: 97.1%; TNBC: 98.6%) (**Table 2**). In the TNBC group, 53.5% of patients with non-pCR received capecitabine as intensive therapy after definitive surgery and adjuvant therapy. In the HR-low/HER2-negative BC group, all patients received adjuvant endocrine therapy, mainly toremifene and tamoxifen.

**Table 2:** Treatment characteristics of the patients^a^.

Characteristic	Low-HR/HER2-negative	TNBC	*P*
	N=70 (%)	N=218 (%)	
NAC treatment cycles			
<6	24 (34.3)	76 (34.9)	1.000
≥6	46 (65.7)	142 (65.1)	
NAC regimen			
TAC	27 (38.6)	75 (34.4)	0.407
AC-T	39 (55.7)	119 (54.6)	
Other	4 (5.7)	24 (11.0)	
Platinum-based NAC regimen			
No	61 (87.1)	179 (82.1)	0.424
Yes	9 (12.9)	39 (17.9)	
Breast surgery			
Breast-conserving surgery	7 (10.0)	8 (3.7)	0.078
Mastectomy	63 (90.0)	210 (96.3)	
Axilla surgery			
ALND	68 (97.1)	215 (98.6)	0.765
SLNB	2 (2.9)	3 (1.4)	

a Low-HR/HER2-negative, low hormone receptor/human epidermal growth factor receptor 2 negative; TNBC, triple-negative breast cancer; NAC, neoadjuvant chemotherapy; ALND, axillary lymph node dissection; SLNB, sentinel lymph node biopsy.

### Efficacy analysis

**Figure 2:**
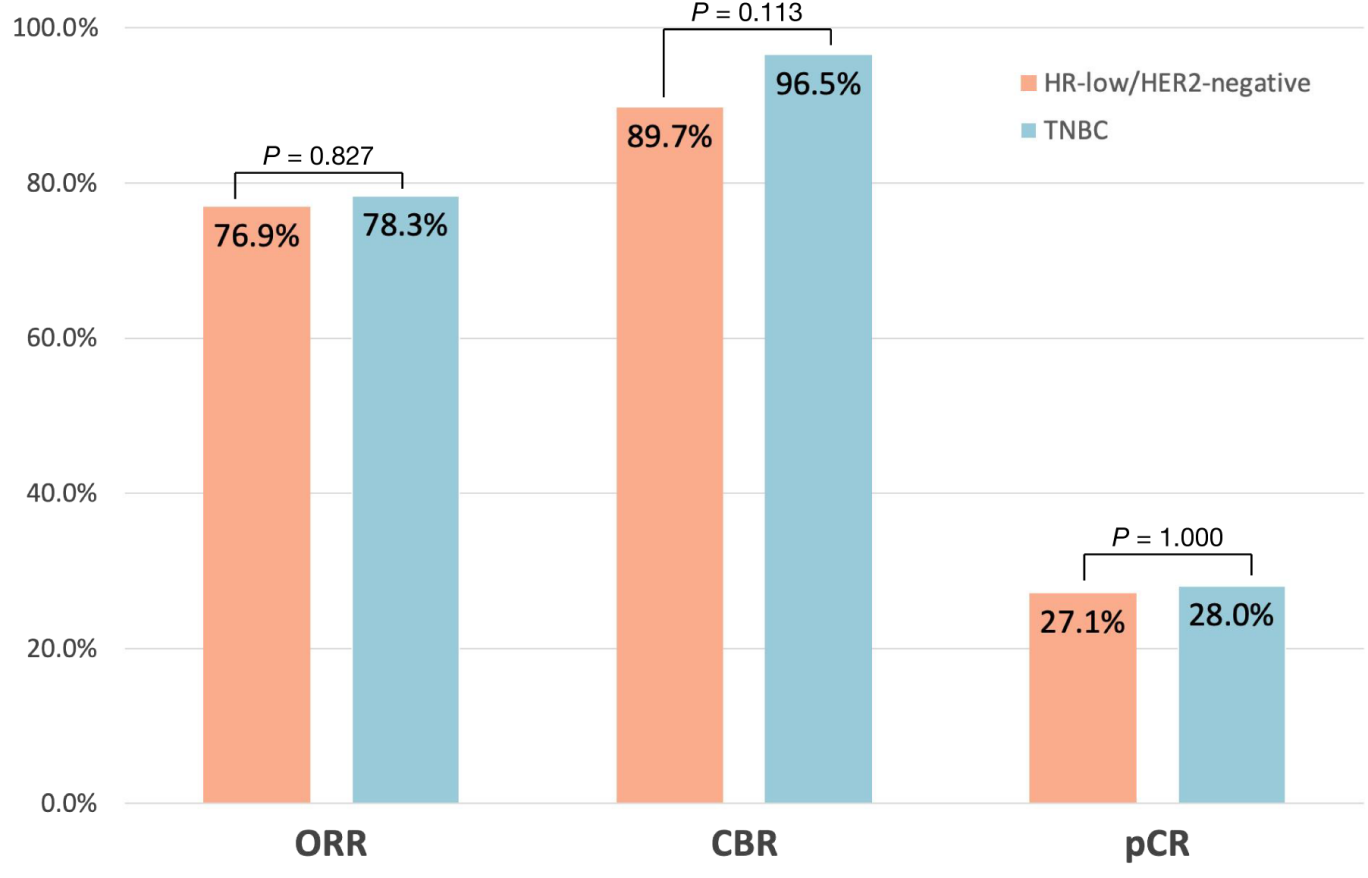
Pathological complete response (pCR: ypT0/is ypN0), objective response rate (ORR), and clinical benefit rate (CBR) across the HR-low/HER2-negative BC group and TNBC group.

The ORR was 76.9% and 78.3% in the HR-low/HER2-negative BC group and the TNBC group, respectively. A total of 89.7% of patients with HR-low/HER2-negative BC and 96.5% of patients with TNBC met the CBR criteria. The pCR rate of the HR-low/HER2-negative BC group was similar to that of the TNBC group (27.1% vs. 28.0%). No significant difference was found in the ORR, CBR, or pCR rate between the 2 groups (ORR: *P* = 0.827; CBR: *P* = 0.113; pCR: *P* = 1.000) (**Figure 2**).

According to the multivariate logistic regression analysis, NAC treatment cycles (*P* = 0.022) remained independently associated with pCR. The pCR rates in the HR-low/HER2-negative BC and TNBC groups were not significantly different according to either univariate or multivariate logistic analysis (**Supplementary Table 1**). According to the multivariate COX regression analysis, pathological nodal status (*P* = 0.001), histological tumour type (*P* = 0.009), and pCR (*P* = 0.013) were independently associated with EFS (**Supplementary Table 2**).

**Figure 3:**
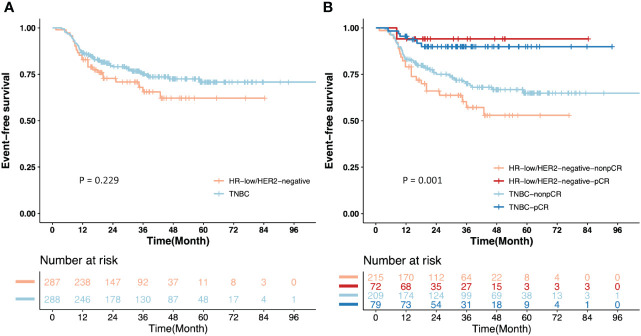
Kaplan-Meier estimates of event-free survival (EFS) after inverse probability of treatment weighting (IPTW). **(A)** EFS of the patients from the HR-low/HER2-negative BC and TNBC groups; **(B)** EFS of the patients with pCR or non-pCR from the HR-low/HER2-negative BC and TNBC groups.

Comparing the EFS (*P* = 0.229) and OS (*P* = 0.579) between the 2 groups after IPTW, no significant difference was found (**Figure 3A**; **Supplementary Figure 1**). The 3-year EFS were 66.50% (95% CI = 54.83% to 80.66%) in the HR-low/HER2-negative BC group and 75.03% (95% CI = 69.15% to 81.41%) in the TNBC group.

Given that pCR is an important survival-relevant factor, additional survival analyses were performed among patients with pCR or non-pCR from the two groups separately. Non-pCR patients in the two groups showed a significantly higher risk of events than those with pCR (*P* = 0.001). The 3-year EFS of the patients with pCR and non-pCR were 94.74% (95% CI = 85.21% to 100.00%) and 57.39% (95% CI =43.81% to 75.19%), respectively, in the HR-low/HER2-negative BC group, and 89.70% (95% CI = 82.20% to 97.90%) and 69.73% (95% CI = 62.51% to 77.77%), respectively, in the TNBC group. For non-pCR patients, the 3-year EFS in the HR-low/HER2-negative BC group was lower than that in the TNBC group (57.39% vs. 69.73%). (**Figure 3B**).

## Discussion

BC is a heterogeneous disease, and distinct subtypes of BC exhibit different treatment sensitivities. HR-low/HER2-negative BCs account for only 6% of all BCs (27). However, the similarity of the biological behaviour of HR-low/HER2-negative BCs and TNBCs has been described multiple times in recent years (6, 14, 28, 29).

Molecular profiling studies have reported that despite the majority of TNBCs being categorized as basal-like BCs, TNBCs contain other molecular subtypes, such as luminal BCs (30–32). It has also been reported that 1-10% of ER-positive tumours are heterogeneous and crossover with not only luminal A/B but also HER2-enriched and basal-like (14, 31–34), indicating a mixed distribution of molecular subtypes in HR-low/HER2-negative BC by IHC.

By comparing the data from our centres, we found that the proportion of TNBC tumours was approximately 3 times that of HR-low/HER2-negative BC tumours, which is consistent with the statistics from prior studies (9, 30). TNBCs generally exhibit a noticeably higher pCR rate than luminal tumours (35–38). However, we found that patients with HR-low/HER2-negative BCs (27.1%) and TNBCs (28.0%) had comparable pCR rates after NAC (*P* = 1.000). The low level of pCR rates in both groups are consistent with those in other real-world studies of the efficacy of NAC in TNBC (39–41), and may be related to patient compliance and differences in drug regimen selection between randomized controlled trials and real-world studies. Additionally, the grouping of HR-low/HER2-negative BCs or TNBCs had no impact on pCR in univariate (*P* = 0.524) and multivariate (*P* = 0.823) analyses of pCR.

In respect of EFS (*P* = 0.229) and OS (*P* = 0.579), no significant difference was found between the HR-low/HER2-negative BC group and the TNBC group (**Figure 3A**; **Supplementary Figure 1**). This outcome may be related to the relatively limited follow-up duration. However, EFS in patients with non-pCR from the two groups was significantly worse than those with pCR (*P* = 0.001). The HR-low/HER2-negative BC group had a lower 3-year EFS than the TNBC group (66.50% vs. 75.03%), and the patients with non-pCR from the HR-low/HER2-negative BC and TNBC groups showed an inferior 3-year EFS to that of the patients with pCR from the two groups (HR-low/HER2-negative BC with pCR and non-pCR: 94.74% vs. 57.39%; TNBC with pCR and non-pCR: 89.70% vs. 69.73%) (**Figure 3B**). No significant difference was observed in the 3-year EFS, but numerical differences did exist. We infer these may be attributed to the application of adjuvant intensive therapy, which remarkably improved the survival outcomes in non-pCR TNBC patients (20). Specifically, we propose that HR-low/HER2-negative BC patients, especially those with non-pCR after NAC, exhibit unmet treatment needs. This observation underscores their potential for intensified adjuvant therapy, such as combinations of capecitabine and endocrine therapy. We highlight the necessity for further prospective randomized controlled clinical studies to confirm these findings.

In addition to the pCR rate, the ORR and CBR are also commonly used therapeutic sensitivity parameters. To our knowledge, this is the first study to estimate ORR and CBR for patients with HR-low/HER2-negative BCs and TNBCs after NAC; likewise, no statistically significant difference was detected between the two subgroups (ORR: *P* = 0.827; CBR: *P* = 0.113; pCR: *P* = 1.000). Taken together, the absence of differences in patients’ baseline characteristics and NAC response between the 2 phenotypes highlights the fact that HR-low/HER2-negative BC appears to be biologically similar to TNBC. Current findings underscore the substantial unmet treatment needs of HR-low/HER2-negative BC patients, particularly those who fail to achieve a pCR following neoadjuvant chemotherapy. The potential efficacy of intensified adjuvant therapies, notably the combination of capecitabine and endocrine therapy, has emerged as a crucial area for addressing these needs. Such an approach could potentially improve outcomes for these patients, underlining the need for a more tailored therapeutic strategy based on HR and HER2 status. To validate these preliminary findings and fully understand the implications for treatment protocols, further prospective randomized controlled clinical studies are warranted.

This study had several limitations. First, this was a multicentre retrospective study, which may have been biased by the completeness of data collection and the accuracy of follow-up information. For example, there may have been slight differences in the data recording standards of different centres, and some prognostic follow-up information, which is derived from patient recollection, may have contained errors. These potentially compromised the accuracy and statistical power of the results. Second, the sample size was relatively small, and the follow-up time was relatively limited, to some extent, possibly posing challenges related to limited statistical ability and the capability to identify significant differences. However, HR-low/HER2-negative BC is indeed a rare subtype, which inherently limits the availability of a larger cohort for analysis. Despite these limitations, our preliminary findings contributed to a deeper understanding of HR-low/HER2-negative BC, and future studies are needed to validate and expand our findings through more rigorous data verification processes, larger cohorts, and extended follow-up periods.

## Conclusions

In summary, the real-world therapeutic effects of NAC for HR-low/HER2-negative BCs and TNBCs were similar. EFS of patients with non-pCR in the HR-low/HER2-negative BC group was inferior to that of the TNBC group with non-pCR, suggesting that it is necessary to explore new adjuvant intensive therapy strategies for HR-low/HER2-negative BC patients.

## Data availability statement

The original contributions presented in the study are included in the article/**Supplementary Material**. Further inquiries can be directed to the corresponding authors.

## Ethics statement

The studies involving humans were approved by the Ethics Committee of the Second Xiangya Hospital of Central South University. The studies were conducted in accordance with the local legislation and institutional requirements. Written informed consent for participation was not required from the participants or the participants’ legal guardians/next of kin in accordance with the national legislation and institutional requirements.

## Author contributions

WY: Conceptualization, Data curation, Funding acquisition, Project administration, Supervision, Writing – review & editing. JP: Data curation, Formal analysis, Investigation, Software, Writing – original draft. YH: Data curation, Methodology, Supervision, Visualization, Writing – review & editing. QC: Data curation, Resources, Validation, Writing – review & editing. FX: Data curation, Resources, Writing – review & editing. DZ: Data curation, Resources, Writing – review & editing. JY: Data curation, Resources, Writing – review & editing. QZ: Data curation, Resources, Writing – review & editing. LY: Data curation, Resources, Writing – review & editing. LL: Data curation, Resources, Writing – review & editing. QL: Data curation, Resources, Writing – review & editing. LL: Data curation, Resources, Writing – review & editing. ML: Data curation, Resources, Writing – review & editing. XL: Data curation, Resources, Writing – review and editing. SW: Conceptualization, Data curation, Project administration, Supervision, Writing – review & editing.

## Supplementary material

Supplementary Figure 1 Kaplan-Meier estimates of overall survival (OS) of the patients from the HR-low/HER2-negative BC and TNBC groups after inverse probability of treatment weighting (IPTW).
