# Supplementary figures and images for: Comparison of neoadjuvant chemotherapy response and prognosis between HR-low/HER2-negative BC and TNBC: an exploratory real-world multicentre cohort study
Source: Front Endocrinol (Lausanne). 2024 Mar 19;15:1347762. doi: 10.3389/fendo.2024.1347762 (PMC10985142; doi:10.3389/fendo.2024.1347762)

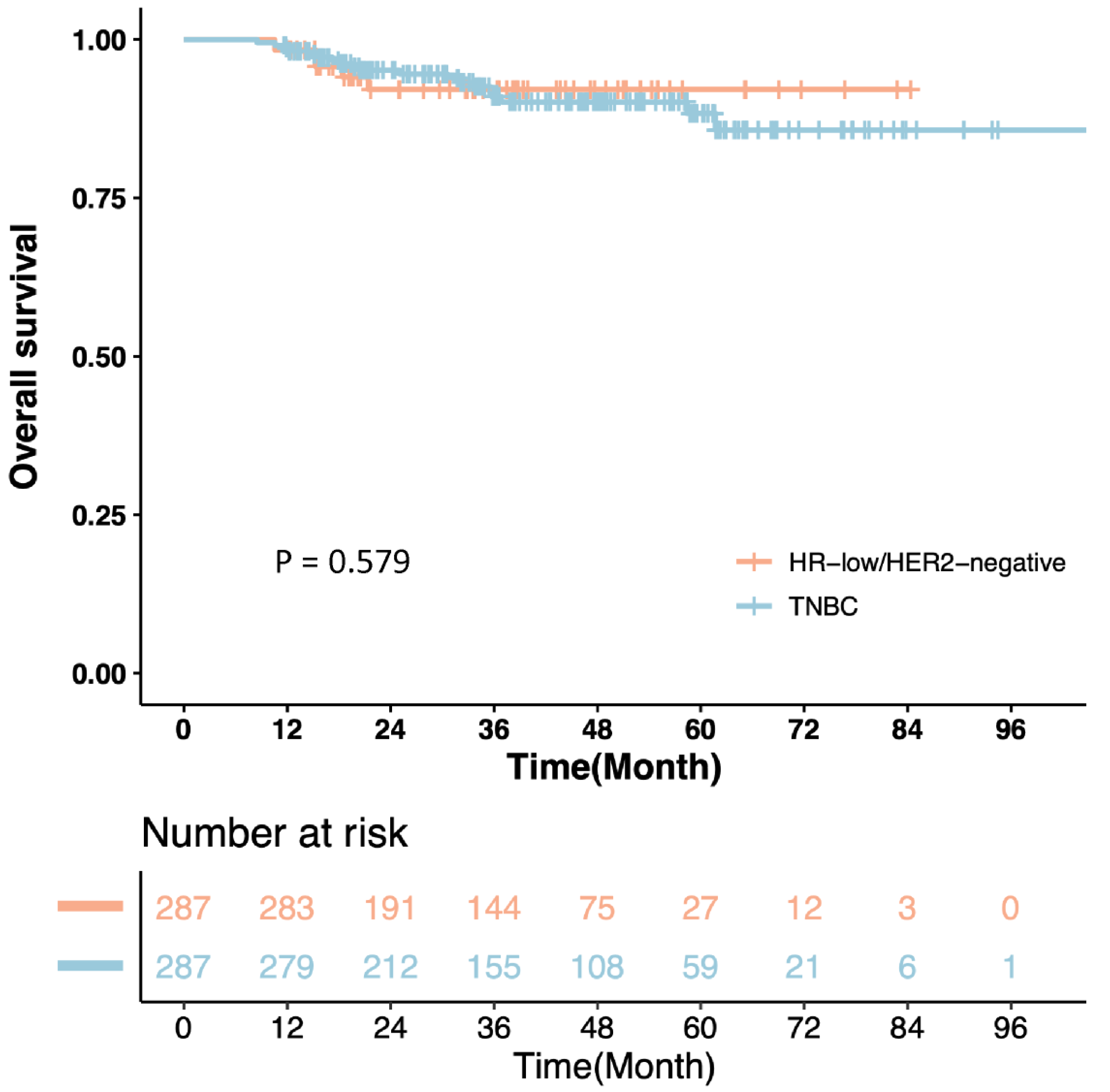

Supplement: Supplementary Figure 1 — Kaplan-Meier estimates of overall survival (OS) of the patients from the HR-low/HER2-negative BC and TNBC groups after inverse probability of treatment weighting (IPTW). [file Image_1.tif]
