# Supplementary material for: Comparison of neoadjuvant chemotherapy response and prognosis between HR-low/HER2-negative BC and TNBC: an exploratory real-world multicentre cohort study
Source: Front Endocrinol (Lausanne). 2024 Mar 19;15:1347762. doi: 10.3389/fendo.2024.1347762 (PMC10985142; doi:10.3389/fendo.2024.1347762)
Supplement: Supplementary file 2 [file Table_1.doc]

Supplementary Table 1. Univariate and multivariate logistic regression analysis of pCR^a^

| Characteristic | Univariate | | Multivariate | |
| --- | --- | --- | --- | --- |
|  | OR (95% CI) | p | OR (95% CI) | p |
| Subtype |  |  |  |  |
| TNBC | 1 |  |  |  |
| Low-HR/HER2-negative | 0.89 (0.61 to 1.28) | 0.524 | 0.93 (0.50 to 1.73) | 0.823 |
| Menopausal status |  |  |  |  |
| Postmenopausal | 1 |  | - | - |
| Premenopausal | 1.02 (0.69 to 1.49) | 0.933 | - | - |
| Clinical tumor stage |  |  |  |  |
| T1-2 | 1 |  | 1 |  |
| T3-4 | 0.44 (0.28 to 0.68) | <0.001 | 0.64 (0.36 to 1.15) | 0.137 |
| Histological tumor type |  |  |  |  |
| IDC | 1 |  | - | - |
| Other | 0.52 (0.25 to 1.08) | 0.080 | - | - |
| Tumor grade |  |  |  |  |
| II | 1 |  | - | - |
| III | 1.38 (0.93 to 2.05) | 0.106 | - | - |
| Unknown | 0.53 (0.27 to 1.06) | 0.073 | - | - |
| NAC treatment cycles |  |  |  |  |
| <6 | 1 |  | 1 |  |
| ≥6 | 2.21 (1.45 to 3.36) | <0.001 | 2.05 (1.11 to 3.79) | 0.022 |
| NAC regimen |  |  |  |  |
| TAC | 1 |  | 1 |  |
| AC-T | 2.01 (1.33 to 3.04) | <0.001 | 1.32 (0.73 to 2.40) | 0.356 |
| Other | 0.95 (0.43 to 2.11) | 0.902 | 1.21 (0.44 to 3.29) | 0.714 |
| Platinum-based NAC regimen |  |  |  |  |
| No | 1 |  | - | - |
| Yes | 0.78 (0.45 to 1.33) | 0.357 | - | - |

^a^ OR = odds ratios, CI = confidence intervals, TNBC = triple-negative breast cancer, Low-HR/HER2-negative = low hormone receptor/human epidermal growth factor receptor 2 negative, NAC = neoadjuvant chemotherapy.
