# Supplementary material for: Comparison of neoadjuvant chemotherapy response and prognosis between HR-low/HER2-negative BC and TNBC: an exploratory real-world multicentre cohort study
Source: Front Endocrinol (Lausanne). 2024 Mar 19;15:1347762. doi: 10.3389/fendo.2024.1347762 (PMC10985142; doi:10.3389/fendo.2024.1347762)
Supplement: Supplementary file 3 [file Table_2.doc]

Supplementary Table 2. Univariate and multivariate Cox regression analysis of EFS^a^

| Characteristic | Cox Regression EFS | | | |
| --- | --- | --- | --- | --- |
|  | Univariate | | Multivariate | |
|  | HR (95% CI) | p | HR (95% CI) | p |
| Subtype |  |  |  |  |
| TNBC | 1 |  | 1 |  |
| Low-HR/HER2-negative | 1.41 (0.84 to 2.35) | 0.189 | 1.32 (0.79 to 2.22) | 0.287 |
| Menopausal status |  |  |  |  |
| Postmenopausal | 1 |  | - | - |
| Premenopausal | 1.33 (0.77 to 2.30) | 0.303 | - | - |
| Clinical tumor stage |  |  |  |  |
| T1-2 | 1 |  | - | - |
| T3-4 | 1.42 (0.82 to 2.48) | 0.214 | - | - |
| Pathological nodal status |  |  |  |  |
| N0 | 1 |  | 1 |  |
| N+ | 6.27 (2.72 to 14.45) | <0.001 | 3.89 (1.91 to 7.93) | 0.001 |
| Histological tumor type |  |  |  |  |
| IDC | 1 |  | 1 |  |
| Other | 2.98 (1.52 to 5.85) | 0.002 | 3.40 (1.35 to 8.56) | 0.009 |
| Tumor grade |  |  |  |  |
| II | 1 |  | 1 |  |
| III | 1.13 (0.62 to 2.04) | 0.691 | 0.85 (0.49 to 1.47) | 0.554 |
| Unknown | 2.15 (1.03 to 4.48) | 0.041 | 0.71 (0.27 to 1.81) | 0.468 |
| NAC treatment cycles |  |  |  |  |
| <6 | 1 |  | - | - |
| ≥6 | 0.72 (0.41 to 1.26) | 0.248 | - | - |
| NAC regimen |  |  |  |  |
| TAC | 1 |  | 1 |  |
| AC-T | 0.49 (0.28 to 0.87) | 0.015 | 0.70 (0.43 to 1.16) | 0.167 |
| Other | 0.81 (0.39 to 1.70) | 0.580 | 1.32 (0.65 to 2.66) | 0.444 |
| Platinum-based NAC regimen |  |  |  |  |
| No | 1 |  | - | - |
| Yes | 1.18 (0.61 to 2.27) | 0.620 | - | - |
| pCR |  |  |  |  |
| No | 1 |  | 1 |  |
| Yes | 0.21 (0.08 to 0.54) | 0.001 | 0.36 (0.16 to 0.81) | 0.013 |

^a^ EFS = event-free survival, HR = hazard ratio, CI = confidence interval, TNBC = triple-negative breast cancer, Low-HR/HER2-negative = low hormone receptor/human epidermal growth factor receptor 2 negative, NAC = neoadjuvant chemotherapy, pCR = pathologic complete response.
